# Ictal source imaging and electroclinical correlation in self-limited epilepsy with centrotemporal spikes

**DOI:** 10.1016/j.seizure.2017.09.006

**Published:** 2017-11

**Authors:** Jørgen Alving, Martin Fabricius, Ivana Rosenzweig, Sándor Beniczky

**Affiliations:** a Department of Clinical Neurophysiology, Danish Epilepsy Center, Dianalund, Denmark; b Department of Clinical Neurophysiology, Copenhagen University Hospital, Rigshospitalet, Copenhagen, Denmark; c Sleep and Brain Plasticity Centre, Department of Neuroimaging, IoPPN, King’s College London & Sleep Disorders Centre, Guy’s and St Thomas’ Hospital, GSTT, London, United Kingdom; d Department of Clinical Neurophysiology, Aarhus University, Aarhus, Denmark

**Keywords:** EEG, Ictal source imaging, Seizure semiology, Self-limited epilepsy with centrotemporal spikes

## Abstract

**Purpose:**

To elucidate the localization of ictal EEG activity, and correlate it to semiological features in self-limited epilepsy with centrotemporal spikes (formerly called “benign epilepsy with centrotemporal spikes”).

**Methods:**

We have performed ictal electric source imaging, and we analysed electroclinical correlations in three patients with self-limited epilepsy with centrotemporal spikes.

**Results:**

The source of the evolving rhythmic ictal activity (9.7-13.5 Hz) localized to the operculo-insular area. The rhythmic EEG activity was time-locked to the contralateral focal motor seizure manifestation: facial rhythmic myoclonic jerks, with the same frequency as the analysed ictal rhythm. In all three patients, the seizures had fluctuating course with pauses of clinical and electrographic seizure activity, ranging from 0.4 to 7 s.

**Conclusion:**

Source imaging of ictal EEG activity in patients with self-limited epilepsy with centrotemporal spikes showed activation of the operculo-insular area, time-locked to the contralateral focal myoclonic jerks. Fragmented seizure dynamics, with fluctuating course, previously described as a hallmark in patients with psychogenic non-epileptic seizures, can occur in rolandic seizures.

## Highlights

• We analysed seizures in patients with self-limited epilepsy with centrotemporal spikes.
• Ictal source imaging showed activation of the operculo-insular area.
• Ictal EEG activity was time-locked to contralateral facial myoclonic jerks.
• The dynamics of the seizures was fragmented.

## 1 Introduction

Self-limited epilepsy with centrotemporal spikes [1] (formerly called “benign epilepsy with centrotemporal spikes” or “idiopatic/benign rolandic epilepsy of childhood”) is the most common syndrome of idiopathic focal epilepsy in children [2]. Seizures typically occur during sleep or awakening. Since seizure frequency is usually low, there are scarce reports on the ictal EEG pattern in this syndrome; most of the papers are case reports or small case series.

The largest number of patients was reported by Capovilla and co-workers [3]. They retrospectively collected 30 patients with ictal recordings, and they identified four types of ictal patterns, the most common being the low-voltage fast activity. However, the precise anatomic location of the ictal activity, and the temporal correlation between the ictal EEG and the semiological manifestation have not been systematically addressed yet.

Source imaging of ictal EEG activity faces additional challenges, due to artefacts which are often superimposed on the ictal EEG activity and due to the rapid propagation [4]. However, advances in signal analysis have made it possible to develop standardised methods for ictal EEG source imaging, that were validated in patients with therapy-resistant focal epilepsy who underwent surgery [4], [5], [6].

Here, we report ictal EEG source imaging and electroclinical correlation in three patients with self-limited epilepsy with centrotemporal spikes. To the best of our knowledge, this is the first report on ictal source imaging in this syndrome.

## 2 Methods

### 2.1 Patients

Video-EEG recordings from three patients with self-limited epilepsy with centrotemporal spikes, who had spontaneous seizures during recording, were analysed. All patients were referred to EEG on clinical indications, and all parents gave their informed consent for the recordings and for analysis and post-processing of the recorded data, for scientific purpose. EEGs were recorded using an extended version of the IFCN 10–20 array, with six additional electrodes in the inferior temporal electrode chain (supplementary document 1) [5]. Table 1 summarises the demographic and clinical data of the patients. Diagnosis was based on the ILAE criteria [7].

**Table 1:** Demographic and clinical data of the patients.

	Patient 1	Patient 2	Patient 3
Demographic data	Male, 12-yo	Female, 9-yo	Male, 9-yo
Family history of epilepsy.	None	None	None
Birth	Caesarian section; no perinatal complications.	Uneventful	Uneventful
Motor and cognitive development	Normal	Normal	Normal
Onset of seizures	10-yo	7-yo	7-yo
Seizure frequency	Less than 1/month	Less than 1/year	Less than 1/year
Neurological and cognitive status	Normal	Normal	Normal
Semiology from historical data	Left hemifacial motor seizure, anarthria, occasionally difficult respiration, propagating to left upper limb.	Left focal motor seizures (tonic/clonic).	Left arm clonic jerks, retching, drooling, guttural sounds
Interictal EEG	Right centrotemporal spikes	Right centrotemporal spikes	Right centrotemporal spikes

### 2.2 Data analysis

Ictal source imaging was performed using the method described and validated in previous studies [4], [6]. Briefly: rhythmic ictal activity at the onset of the seizures was identified visually and using density spectral array (DSA). The initial part of the rhythmic activity was defined using Fast Fourier Transform in sliding windows with 50% overlap (allowing for gradual change of ± 1 Hz), independent component analysis (ICA), and inspection of the voltage map of each ictal wave. Averaged ictal waveforms were analysed using two different inverse methods: discrete multiple dipoles fitting, and a distributed source model in the brain volume, i.e., classical LORETA analysis recursively applied (CLARA) [6]. As head model, we used a finite-elements model (FEM) in BESA-MRI, with age-matched templates. BESA Research 6.1 was used for the signal analysis.

Video-recordings were analysed and reported by three trained clinical neurophysiologists, with more than 10-year experience in long-term video-EEG monitoring.

## 3 Results

The EEG pattern showed similar features in all three patients. In the period preceding the seizures, an increase in the occurrence of right centrotemporal sharp-and-slow wave discharges was observed, leading to quasi-rhythmic trains of 1–2 Hz frequency. In patient 2, a second pre-ictal focus of sharp-and-slow-waves occurred in the left central and mid-central area. Two to ten seconds before the start of the clinical seizure, the pre-ictal sharp-and-slow-wave activity was replaced in the right centrotemporal region by evolving rhythmic ictal activity, starting with low-amplitude, 9.7–13.5 Hz frequency (supplementary document 1), and gradually increasing in amplitude and decreasing in frequency to 6–8 Hz.

**Fig. 1:**
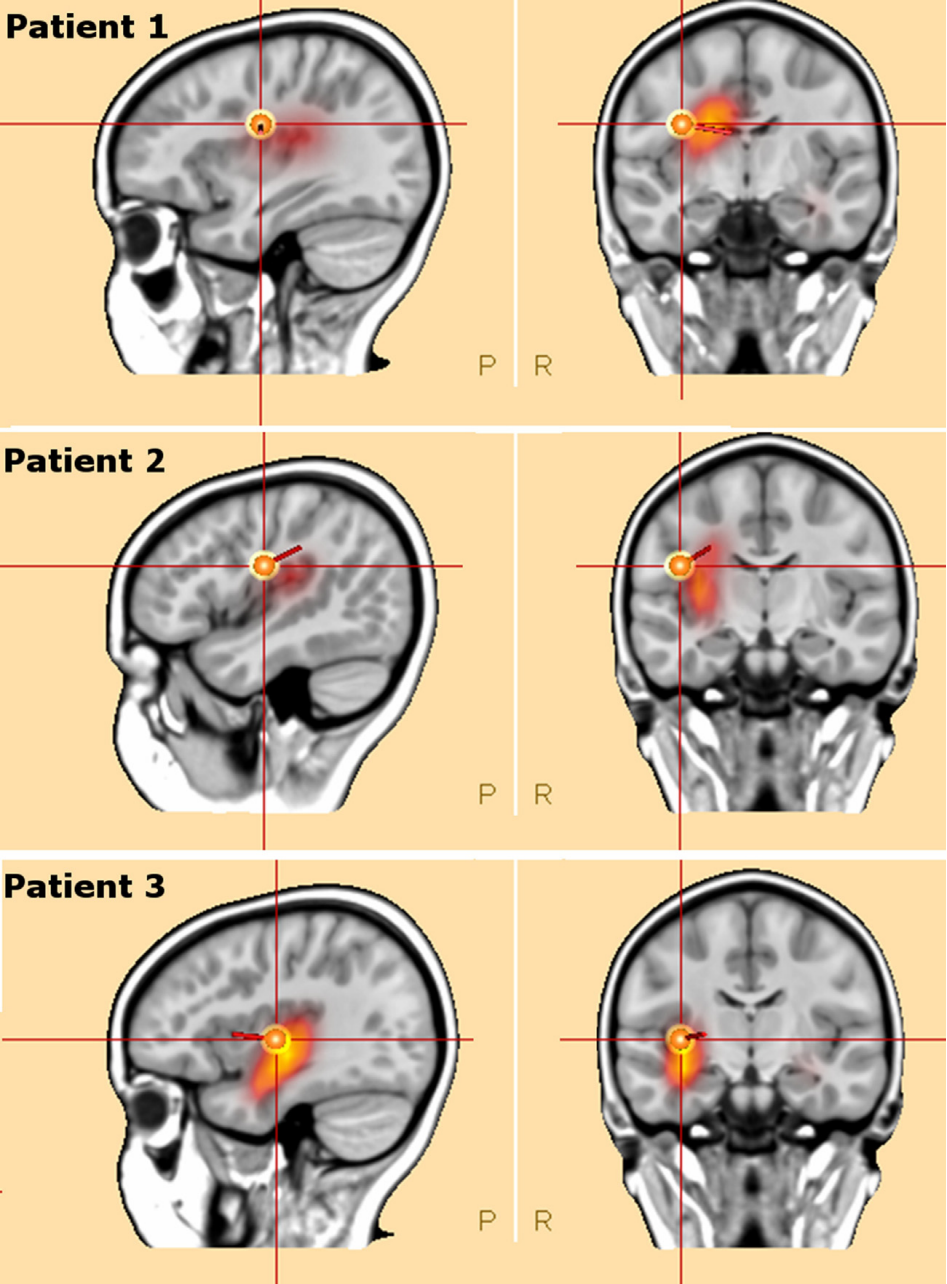
shows ictal source imaging using dipole and distributed source model (CLARA) in sagittal and coronal slices in the three patients.

Source imaging of the ictal activity localized to the right operculo-insular area (Fig. 1). Equivalent current dipoles localized to the opercular part of the right central area, with exception of the ictal activity in patient 3, where it was localized to the insula. Distributed source model localized to the right insula in all three cases. In patient 2, the second (contralateral) pre-ictal focus localized to the left mesial central area.

Seizures started from the awake period in one patient, from drowsiness in the second patient and immediately following awakening in the third patient. EEG start preceded clinical start in all three cases. The first semiological manifestation was left perioral: tonic muscle activation causing mouth-deviation to the left. This was followed by arrhythmic myoclonic jerks, superimposed on the focal tonic muscle activation. These phenomena gradually extended from the perioral region to the left side of the face. The myoclonic jerks increased in frequency, became rhythmic at 9.5 Hz (patient 1), 8.1 Hz (patient 2) and 8.2 Hz (patient 3). These high frequency clonic jerks were time-locked to the contralateral EEG rhythmic activity with the same frequency.

The seizures were fragmented, and they showed a fluctuating course with pauses of ictal activity in all three cases. The pauses consisted in total cessation of clinical and electrographic seizure activity, ranging from 0.4 to 7 s (patient 1: 0.6 s; patient 2: 2 s; patient 3: 26 short stop periods of 0.4 to 2 s duration and a long period of 7 s). Duration of their respective seizures was 33 s in patient 1, 45 s in patient 2, and 2 min and 21 s in patient 3.

## 4 Discussion

Previous studies using electromagnetic source imaging have predominantly analysed the location of the interictal epileptiform discharges in self-limited epilepsy with centrotemporal spikes [8]. These studies reported sources of the irritative zone to be in the inferior part of the Rolandic area and the operculum [8]. However, it has long been posited that the irritative zone might not be necessarily identical with the area that generates the seizures, and hence the source imaging of ictal activity should be obtained whenever possible.

In this study, we have found that the source of ictal activity was in the operculum and insula. This is consistent with data from intracranial recordings in patients with therapy-resistant focal epilepsy, showing that seizures originating in the opercular rolandic area had semiology similar to patients with self-limited epilepsy with centrotemporal spikes (contralateral facial motor seizures) [9]. Furthermore, the frequency of the ictal rhythms recorded by intracranial electrodes in this area was in the alpha and lower beta range [9], which is similar to the ictal rhythms we analysed in this study. The contralateral myoclonic jerks in our patients were time-locked to the rhythmic ictal activity we analysed, underlying the correlation between the observed activity in the operculo-insular area and the semiological phenomena. In keeping with our findings, a previous study using fluorodeoxyglucose-positron emission tomography (FDG-PET) also showed significant changes in the opercular areas in self-limited epilepsy with centrotemporal spikes [10]. In addition, a study combining EEG source imaging and fMRI showed propagation of the interictal activity from the rolandic region corresponding to the hand and face area, to the operculum and insula [11]. It has been previously suggested that involvement of insula likely explains the sensations of laryngeal constriction and choking that is often reported by patients with self-limited epilepsy with centrotemporal spikes [3].

It is of particular note that in our patients a fluctuating, fragmented course was recorded, with complete pauses of ictal EEG and motor activity. Similar fluctuating course has previously been described in patients with psychogenic non-epileptic seizures [12]. Our findings suggest that a frank centrotemporal ictal activity might also show intermittent progression. To avoid misdiagnosis, it is important to emphasise that such seizure-dynamics can occur in rolandic seizures too.

To the best of our knowledge, this is the first study on ictal source imaging in patients with self-limited epilepsy with centrotemporal spikes. Our findings emphasise the importance of the operculo-insular network for the ictogenesis in this syndrome.

## Conflict of interest

None of the authors has any conflict of interest to disclose.
