# Supplementary figures and images for: Ictal source imaging and electroclinical correlation in self-limited epilepsy with centrotemporal spikes
Source: Seizure. 2017 Nov;52:7–10. doi: 10.1016/j.seizure.2017.09.006 (PMC5703029; doi:10.1016/j.seizure.2017.09.006)

# Supplementary document 1

Build-up of rhythmic ictal activity. Common average montage

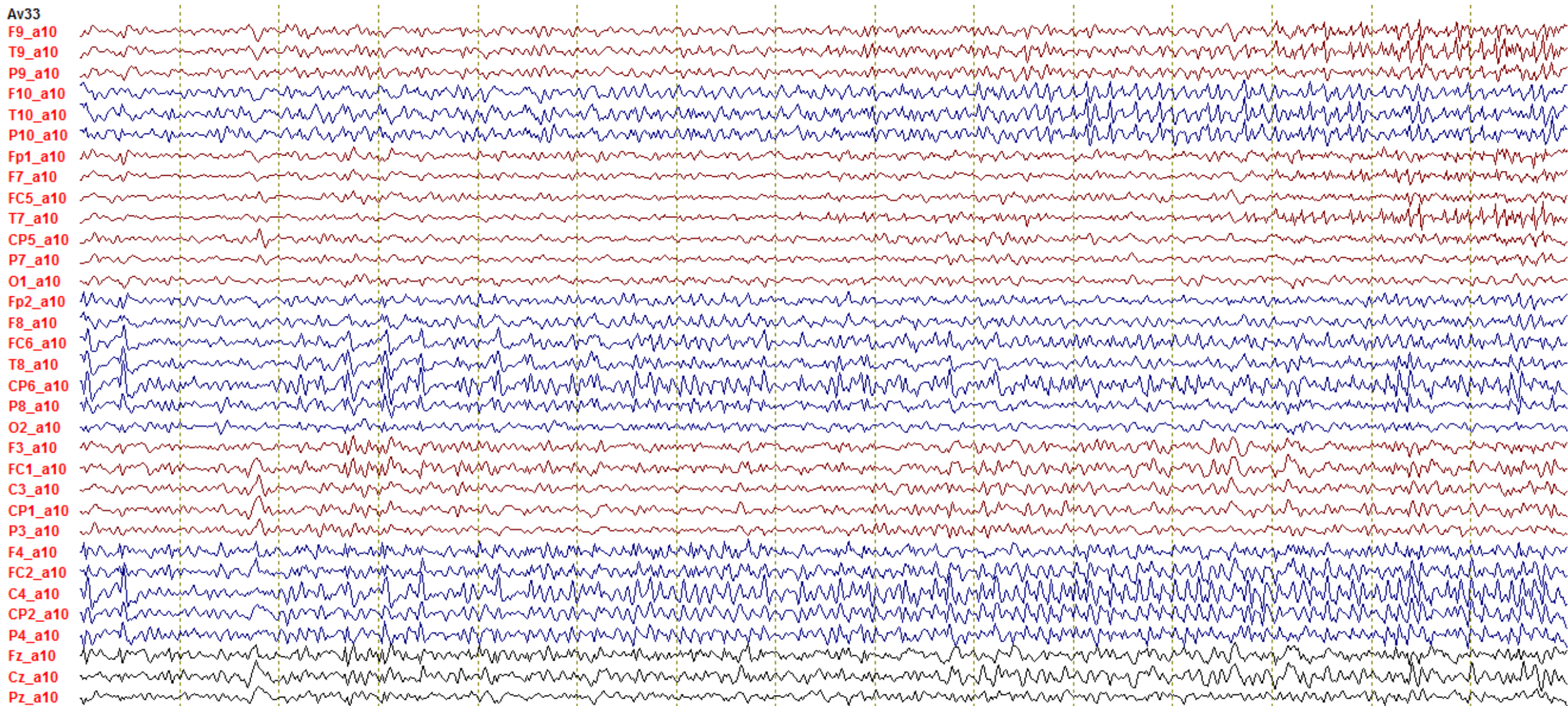

Supplement: Supplementary file 1 [file mmc1.pdf]
